# Economic Evaluation of Provision of Postpartum Intrauterine Device Services in Bangladesh and Tanzania

**DOI:** 10.9745/GHSP-D-20-00447

**Published:** 2021-03-31

**Authors:** Gillian Eva, Judy Gold, Anita Makins, Suzanna Bright, Katherine Dean, Emily-Anne Tunnacliffe, Parveen Fatima, Afroja Yesmin, Projestine Muganyizi, Grasiana F. Kimario, Kim Dalziel

**Affiliations:** a Independent consultant, Washington, DC, USA.; b Independent consultant, Melbourne, Australia.; c International Federation of Gynecology and Obstetrics, London, UK.; d Oxford University Hospitals NHS Foundation Trust, Oxford, UK.; e Nuffield Department Women's and Reproductive Health, Oxford University, Oxford, UK.; f Obstetrical and Gynaecological Society of Bangladesh, Dhaka, Bangladesh.; g Muhimbili University of Health and Allied Sciences, Dar es Salaam, Tanzania.; h Tanzania Midwives Association, Dar es Salaam, Tanzania.; i Melbourne School of Population and Global Health, The University of Melbourne, Melbourne, Australia.

## Abstract

**Introduction:**

Postpartum family planning is an effective means of achieving improved health outcomes for women and children, especially in low- and middle-income settings. We assessed the cost-effectiveness of an immediate postpartum intrauterine device (PPIUD) initiative compared with standard practice in Bangladesh and Tanzania (which is no immediate postpartum family planning counseling or service provision) to inform resource allocation decisions for governments and donors.

**Methods:**

A decision analysis was constructed to compare the PPIUD program with standard practice. The analysis was based on the number of PPIUD insertions, which were then modeled using the Impact 2 tool to produce estimates of cost per couple-years of protection (CYP) and cost per disability-adjusted life years (DALYs) averted. A micro-costing approach was used to estimate the costs of conducting the program, and downstream cost savings were generated by the Impact 2 tool. Results are presented first for the program as evaluated, and second, based on a hypothetical national scale-up scenario. One-way sensitivity analyses were conducted.

**Results:**

Compared to standard practice, the PPIUD program resulted in an incremental cost-effectiveness ratio (ICER) of US$14.60 per CYP and US$91.13 per DALY averted in Bangladesh, and US$54.57 per CYP and US$67.67 per DALY averted in Tanzania. When incorporating estimated direct health care costs saved, the results for Bangladesh were dominant (PPIUD is cheaper and more effective versus standard practice). For Tanzania, the PPIUD initiative was highly cost-effective, with the ICER (incorporating direct health care costs saved) estimated at US$15.20 per CYP and US$18.90 per DALY averted compared to standard practice. For the national scale-up model, the results were dominant in both countries.

**Conclusions/Implications:** The PPIUD initiative was highly cost-effective in Bangladesh and Tanzania, and national scale-up of PPIUD could produce long-term savings in direct health care costs in both countries. These analyses provide a compelling case for national governments and international donors to invest in PPIUD as part of their family planning strategies.

## Abstract (precis)

Provision of a postpartum intrauterine device (PPIUD) within 48 hours of delivery was highly cost-effective compared with standard practice in 2 lower middle-income countries. Policy makers should consider expansion of postpartum family planning counseling and introduction of immediate PPIUD services as an added tool to address the unmet need for contraception.

## Teaser

**Key Findings:**

- Delivering family planning counseling and offering the immediate postpartum intrauterine device (PPIUD) was found to be cost-effective compared to the standard PPFP practice.
- The PPIUD program resulted in an incremental cost-effectiveness ratio of US$14.60 per couple years of protection (CYP) and US$91.13 per disability adjusted life year (DALY) averted in Bangladesh and US$54.57 per CYP and US$67.67 per DALY averted in Tanzania.
- It is likely that national rollout of PPFP counseling and PPIUD delivery will save costs to the health care system in both countries.

**Key Implications:**

- There is a strong case for governments and donors to invest in providing high-quality family planning counseling during antenatal care and around the time of delivery and to include PPIUD within PPFP provision immediately following delivery.
- National provision of PPIUD could produce long-term savings in health care costs due to the decrease in unplanned pregnancies resulting from increased PPFP uptake.
- PPIUD could be even better value if health care providers receive preservice training in this method and if PPIUD delivery was rolled out nationally.

## INTRODUCTION

Postpartum family planning (PPFP) is widely recognized as an important approach to achieving progress towards improved health outcomes for women and children.^1^^–^^3^ The World Health Organization (WHO) advises a minimum of 24 months between a live birth and trying for the next pregnancy^4^ owing to the increased risks to the mother and child of short interpregnancy intervals (the definition of which differs across studies), including miscarriage, induced abortion, stillbirth, preterm birth, low birth weight, infant mortality, and child malnutrition.^5^^–^^9^ Many contraceptive methods are now considered safe to use postpartum, even among breastfeeding women.^10^^,^^11^ In addition, the increasing number of women in low- and middle- income countries (LMICs) attending antenatal care and delivering in health facilities means that discussing PPFP during antenatal care and at the time of delivery and offering effective postpartum contraception immediately postpartum are now key ways to reduce the risk of unintended pregnancies.^3^^,^^5^^,^^7^^,^^12^

Although many women who give birth do not want another pregnancy within 12 months,^13^ births at short interpregnancy intervals are not uncommon, especially in LMICs.^14^ PPFP use remains low and is mostly unchanged over the last decade, particularly across Africa,^15^ resulting in high unmet need among postpartum women for both spacing and limiting births.^13^^,^^14^^,^^16^ Institutional delivery and child immunization are the factors most correlated with voluntary uptake of modern PPFP,^15^^,^^17^ and several studies have demonstrated the importance of good-quality counseling and community involvement to increasing PPFP acceptance.^18^^–^^20^ Challenges identified for PPFP uptake include perceived low risk of pregnancy during the postpartum period among both providers and women, low rates of facility deliveries, and (perceived or real) cultural resistance to family planning, particularly during the postpartum period.^2^^,^^21^ The latest WHO Medical Eligibility Criteria (MEC) guidance in 2015 included several additional methods that can be initiated immediately postpartum.^10^ Before this guideline change, PPFP was often not discussed until the 6-week follow-up visit, which many women do not attend and which comes after the return of fertility for women who are not exclusively breastfeeding.

Long-acting reversible contraceptives (LARCs) have the potential to be an important component of PPFP programs, especially because they have very low failure rates, do not require resupply visits, and can be reversed. Women who do use PPFP mostly use short-acting methods, and very few use a postpartum intrauterine device (PPIUD).^13^^,^^14^ The WHO MEC guidance states that long-acting methods (intrauterine devices [IUDs], intrauterine systems (IUSs), and implants), can be used immediately postpartum.^10^ They are also appropriate for breastfeeding women; IUDs can be used without restrictions, and implants and IUSs are methods for which the advantages of use generally outweigh the risks.^10^ IUSs and implants have high costs, which means that IUSs are rarely available in LMICs, and the availability of implants frequently depends on subsidies or donor supplies rather than national government purchasing.^22^^–^^24^

The copper IUD has been available in both LMICs and high-income countries for decades as an interval method (after 6 weeks postpartum), but it has not been routinely used immediately postpartum.^25^ Provision of immediate PPIUD leads to a lower risk of future unintended pregnancies and higher continued use at 6 months, compared with IUD provided at a later time.^26^ Although previous studies reported higher expulsion rates for immediate PPIUD compared with insertions at other times,^26^^–^^28^ 2 recent studies showed that when Kelly forceps are used to ensure correct placement at the fundus of the uterus, expulsion rates of immediate PPIUD insertion are comparable to interval insertion (<5%).^29^^,^^30^ Several programs in both high- and low-income countries have demonstrated that immediate PPIUD is a safe method with low expulsion and discontinuation rates and high acceptance among providers and clients.^11^^,^^27^^,^^31^^–^^34^ Immediate PPIUD also offers cost and time savings to women since they do not have to return to the facility to receive their PPFP method and can combine their follow-up visit with their routine postpartum checkup.

Provision of immediate PPIUD leads to a lower risk of future unintended pregnancies and higher continued use at 6 months, versus IUD provided later.

Recent global efforts have focused on expanding family planning access, including PPFP, through programs such as FP2020.^12^^,^^35^ However, the funding landscape is changing, with uncertainty in the continuity of donor funding and increasing expectation for LMICs to financially sustain their own health services, such as through national health insurance schemes.^36^^,^^37^ Advocating for sufficient investment for widescale provision of PPFP counseling and PPIUD provision is hindered by a limited number of studies and a consequent gap in the evidence base on the cost-effectiveness of these approaches.

The International Federation of Gynecology and Obstetrics (FIGO) conducted a PPIUD initiative between 2013 and 2020 across 6 countries in Africa and Asia. Published analyses from the initiative have demonstrated the feasibility and safety of immediate PPIUD provision, with almost 37,000 PPIUDs inserted between May 2014 and September 2017 in the 6 countries, a low expulsion rate of 2.6% overall, and no cases of uterine perforation.^29^ Our study presents an economic evaluation based on the implementation of the PPIUD initiative in Bangladesh and Tanzania, which was led by FIGO and its national member societies—the Obstetrical and Gynaecological Society of Bangladesh, the Association of Gynaecologists and Obstetricians of Tanzania, and the Tanzanian Midwifery Association. The aim of the evaluation is to inform future national and global efforts to increase access to PPFP counseling and PPIUD provision.

This economic evaluation of the PPIUD initiative in Bangladesh and Tanzania aims to inform efforts to increase access to PPFP counseling and PPIUD provision.

## METHODS

### Target Population, Setting, and Location

The target population was women in Tanzania and Bangladesh attending the facilities participating in the FIGO PPIUD initiative for delivery (6 facilities in each country). All the participating facilities were large tertiary teaching and referral hospitals. In both countries, counseling on postpartum contraception was offered when women were admitted for delivery, as well as during antenatal care at these facilities. In Tanzania, it was also offered during antenatal care at 26 satellite facilities linked to the participating hospitals.

### Intervention (PPIUD Initiative and Context)

The most recent Demographic and Health Surveys (DHS) found that in Bangladesh, 12% of married women of reproductive age have an unmet need for family planning and 52% use modern contraception.^38^ In Tanzania, 22% of married women have an unmet for family planning and just 32% use a modern contraceptive method.^39^ In both countries, less than 1% of women choose to use an IUD (Table 1).^38^^–^^42^ Most women in both countries receive at least one antenatal care visit, and almost half of women in Bangladesh and two-thirds of women in Tanzania deliver at a health facility.

**TABLE 1.** Country Demographic and Health Data^a^

	Bangladesh^38^	Tanzania^39^
2018 population, millions^40^	161.4	56.3
2018 population density, people/km^2^ of land area^40^	1,240	64
Total fertility rate, births per woman	2.3	5.2
Use of modern method of contraception,^b^ %	51.9	32
Family planning uptake at 1–2 months postpartum, %^41^	13.2	9.2^c^
Unmet need for family planning,^b^ %	12.0	22.1
Use of intrauterine device,^b^ %	0.6	0.9
Received antenatal care at least once from a medically trained provider,^d,^^e^ %	81.9	98.0
Delivered at a health facility,^d,^^e^ %	49.4	62.6
Deliveries attended by a skilled provider,^d^ %^42^	52.7	63.6

a Source: Demographic and Health Survey, unless otherwise stated.

b Among currently married women aged 15–49 years.

c Tabulations based on use of family planning obtained from the reproductive calendar (average of use in time span postpartum), births 12–23 months preceding the interview, based on Bangladesh DHS 2011 and Tanzania DHS 2010.

d Among women aged 15–49 years who had a live birth within 3 years of the survey.

e Medically trained providers include qualified doctor, nurse, midwife, family welfare visitor, and community skilled birth attendant. For antenatal care, medically trained providers also include paramedics, medical assistants, or subassistant community medical officer.

The economic evaluation focused on the second phase of the FIGO PPIUD initiative, which ran from January 2015 to June 2018. Full details of the FIGO PPIUD initiative were published previously.^33^ In short, the PPIUD initiative included training on and the provision of PPFP counseling (on all postpartum methods), PPIUD insertion (if eligible and voluntarily chosen), and follow-up at 6 weeks. Each country established a central project team at national professional societies to develop and roll out the PPIUD initiative at 6 large tertiary teaching and referral hospitals. In both countries the national teams consisted of 6 project staff, although not all were employed full-time by the PPIUD project. One facility coordinator and one deputy facility coordinator, both clinicians, oversaw the project at each participating facility in each country.

Based on shared lessons learned among the 6 countries involved in the PPIUD initiative, an initial “training of trainer” session was held, after which all training of trainer and cascade trainings were conducted by national staff on the PPIUD project team. Existing clinical staff at the participating facilities were trained on PPFP counseling and immediate PPIUD insertion. In Bangladesh, 1,160 providers (predominantly doctors) were trained in PPIUD insertion and training lasted 1 day (note this number includes some providers who were trained more than once). Due to the high flow of clients in the Bangladesh facilities, 28 dedicated postpartum contraceptive counselors were also recruited and received an initial 2.5-day training followed by a half-day refresher training the following year. In Tanzania, 1,113 providers received a 3-day PPIUD insertion training, and 1,515 received a 3-day PPFP counseling training. The health care providers trained in PPIUD insertion in Tanzania were a mix of doctors, nurses, and nurse-midwives, and the training content was adapted to suit all cadres and to align with national requirements.

No community-level demand generation activities were included as part of the initiative in these 2 countries, although leaflets and informative videos were produced as an adjunct to counseling in the hospitals as part of the PPIUD initiative. Voluntary insertion of a Copper T 380A IUD was available to any woman who was medically eligible, voluntarily consented to receive an IUD, and attended a PPIUD initiative facility for delivery.

For the initiative and this evaluation, a PPIUD was defined as an IUD inserted immediately following delivery, before the woman was discharged. This could be within 10 minutes of delivery of the placenta (post placental) or between 10 minutes and 48 hours following placental delivery (immediately postpartum).

Ethical approval for the overall FIGO PPIUD initiative was obtained in both countries and from the London School of Hygiene and Tropical Medicine for overall analysis of the data.

### Comparator (Standard Practice)

Standard practice PPFP in both countries was assumed to be no provision of immediate PPFP. The only immediate postpartum contraceptive method available at the facilities during the timeframe of the initiative was tubal ligation during cesarean delivery, which was not routinely available to all women (and very rare in Tanzania). Where PPFP counseling was provided, it typically occurred at the 6-week follow-up postnatal care visit (i.e., outside the defined period of immediate postpartum contraception).

The governments of both countries have expressed official support for increasing access to postpartum contraception, for example, through the 2017 National Action Plan for Family Planning in Bangladesh^43^ and the 2015 Postpartum Family Planning Action Plan and 2019 National Family Planning Costed Implementation Plan in Tanzania.^44^^,^^45^ However, a shortage of trained providers, inconsistent availability of products, and poor infrastructure limit the extent to which these services can be accessed. Immediate PPFP, including provision of IUDs at or around the time of delivery, is not currently standard practice in government health facilities in either country.

Immediate PPFP, including provision of IUDs at or around the time of delivery, is not currently standard practice in government health facilities in Bangladesh or Tanzania.

Although no immediate PPFP (within 48 hours of delivery) is routinely available in either country, PPFP from 6 weeks onwards is offered and it is likely that some of the women who adopted a PPIUD would otherwise have taken up an alternate method during the extended postpartum period. Due to the lack of direct comparators and a lack of available data on uptake of other PPFP in the extended postpartum period, we did not include any alternate methods as the comparator in our main analysis. We have instead included a sensitivity analysis testing the impact of different proportions of women taking up alternate PPFP methods, based on the national uptake rate of PPFP. See the Supplement for full details.

### Economic Evaluation Perspective, Design, and Time Horizon

The economic evaluation involved a decision analysis that compared the new PPIUD initiative with standard practice. A decision analysis was used because it was able to reflect whether women voluntarily accept contraception provided in the immediate postpartum period. The economic evaluation was composed of the incremental costs of the PPIUD initiative (relative to standard practice) and uptake of the PPIUD. This included costs for recruitment; project staff; meetings; equipment; training; development of information, education, and communication materials; clinical supervision; and sharing of data and learning. Full details can be found in the Supplement.

For each country we defined an initial setup period of 4 months; March to June 2015 in Bangladesh and December 2015 to March 2016 in Tanzania. The setup period included 3 months of initial project establishment and 1 month in which the first training of trainers was conducted. The setup period thus included fixed costs but no impact (no PPIUDs inserted). The implementation period, based on the actual timing of the PPIUD initiative, was July 2015 to June 2018 for Bangladesh (36 months) and April 2016 to June 2018 for Tanzania (27 months); the implementation period included ongoing costs of implementation as well as impact (number of PPIUDs inserted).

**FIGURE 1:**
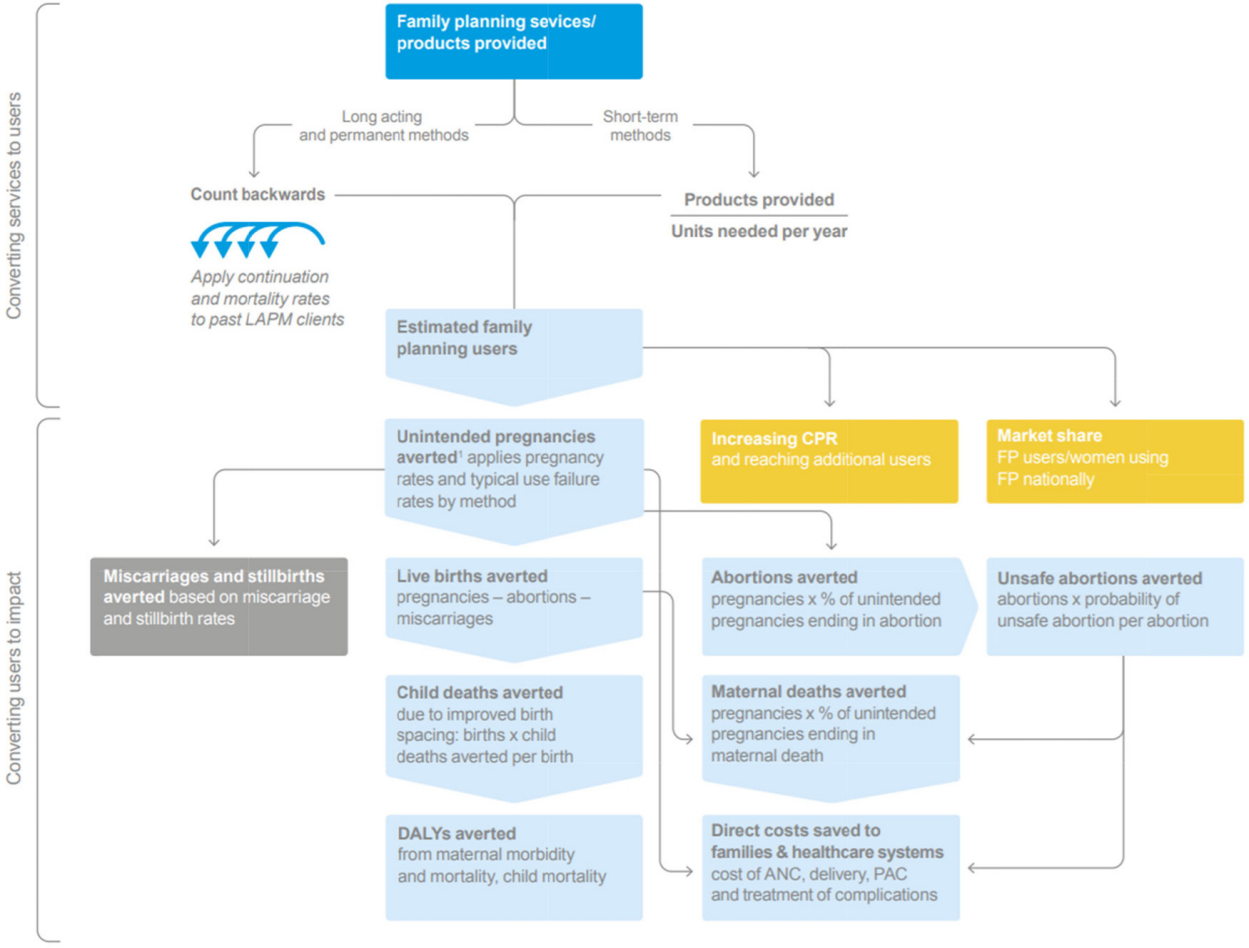
Overview of Impact 2 Tool Used to Assess Cost-Effectiveness of Postpartum Intrauterine Device Initiative Abbreviations: ANC, antenatal care; CPR, contraceptive prevalence rate; DALYs, disability-adjusted life years; FP, family planning; LAPM, long-acting permanent method; PAC, postabortion care; PPIUD, postpartum intrauterine device. Source: Weinberger et al.^47^

The analyses were conducted from the government's perspective. Cost-effectiveness was reported within the time frame of program operation and was also modeled using the existing Impact 2 tool (Figure 1).^46^^,^^47^ In brief, the Impact 2 tool uses national- and regional-level data on typical pregnancy rates and rates of maternal deaths, unsafe abortions, child deaths, and similar outcomes to estimate the impact on key health outcomes of contraceptive services delivered, based on the number of pregnancies and pregnancy-related deaths or illnesses that are averted because a woman is using contraception (Figure 1). The Impact 2 tool also estimates the direct cost savings to the health care system as a result of these health outcomes being averted, based on cost of antenatal care, delivery, postabortion care, and treatment of complications that are averted. The estimated impact of the services will occur over the lifetime of the contraceptive method provided.

The economic analyses were conducted from the government's perspective.

To maximize the usefulness of the evaluation for national governments, we repeated the economic evaluation based on a hypothetical national scale-up. In Bangladesh, we modeled the cost of scaling up the PPIUD initiative to all 36 Government Medical College Hospitals nationally.^48^ In Tanzania, we modeled the cost of scaling up the PPIUD initiative to all 28 Regional Referral Hospitals nationally,^49^ as well as to 140 satellite facilities (assuming an average of 5 per hospital). PPIUD insertion rates for the national scale-up model were based on the insertion rates during the PPIUD initiative. Full details of the adjustments and assumptions made for this analysis can be found in the Supplement.

This manuscript has been prepared in accordance with the Consolidated Health Economic Evaluation Reporting Standards (CHEERS).^50^

### Effectiveness Measures

The measure of effectiveness of the PPIUD initiative was based on the number of immediate PPIUDs inserted, taken directly from the recorded data in the 2 countries, during implementation of the initiative. This measure is relevant for family planning and as an input to the existing Impact 2 tool, which quantifies the relationship between number of insertions, couple-years of protection (CYP), health outcomes, and future costs averted. The primary outcomes for this economic evaluation are cost per PPIUD inserted, cost per CYP,^51^ and cost per disability-adjusted life year (DALY) averted.

### Estimating Resources and Costs

Costs that were provided in local currencies were first adjusted to 2018 local currency costs based on available national inflation data.^52^^,^^53^ The resulting 2018 local currency costs were then converted to US$ using the average exchange rate for the year. Costs that were provided in US$ were adjusted to 2018 US$ using the annual average US inflation rates.^54^ No discount rate was applied to the costs of conducting the PPIUD program or its associated uptake due to the short timeframe of the initiative.

A bottom-up, micro-costing approach was used with inputs as described in the Supplement. Data on costs and PPIUD insertions were primarily sourced from existing project narrative and financial reports, with additional cost data collected as needed from the national project teams. The economic evaluation included the following costs:

- Training of providers in PPIUD insertion and PPFP counseling;
- Staff salary and honorarium payments for facility level staff;
- Reusable clinical equipment;
- Lifetime direct PPIUD service delivery costs: cost of insertion, follow-up visit (if any), and removal; and
- Costs of supporting activities: behavior change materials, advocacy, project management, and monitoring.

A 10% overhead rate was applied, as per the overhead rate used by the government in each country. See the Supplement for further information on costs included.

We included costs for all postpartum contraception counseling sessions delivered at the participating facilities during the initiative, regardless of whether the counseled woman adopted a PPIUD, because more women will need to receive counseling than eventually receive a PPIUD. We included costs for people to attend 1 follow-up visit at a health facility, using an attendance rate of 25%, based on follow-up rates achieved during the initiative.

The Government of Bangladesh pays reimbursements for uptake of LARCs; part of the reimbursement is paid to the woman and part to the provider. For IUDs (including PPIUD), up to US$6.24 is available as reimbursement (email communication, July 13, 2020). However, due to administrative challenges, payment of these reimbursements was not consistent during the evaluation timeframe. No reimbursements are paid to women or providers for attendance at follow-up visits in either country.

Costs associated with the initiative being an international, donor-funded project and the costs of the research component of the initiative were excluded from the analysis since these are not reflective of the true cost of government-led introduction of PPIUD. Costs to the women or to society were not included, other than where fees charged to women offset the cost to the government. Consistent with sector standards, costs to treat complications are not included in the analysis.^55^

### Analysis

Incremental cost-effectiveness ratios (ICERs) were generated for the PPIUD initiative as it was conducted in the 6 facilities in each country compared with standard practice using the formula below. The PPIUD initiative was considered as standard postpartum practice *plus* PPFP counseling and PPIUD service delivery, meaning that the cost of standard practice can be estimated as 0 for both the initiative and for standard practice alone. ICERs are reported both with and without the estimated direct health care savings from the Impact 2 tool factored in; when these estimated savings from the Impact 2 tool are factored in, we refer to “ICER with cost offset.”

$$\text{ICER}=\;\;\;\;\;\;\;\;\;\;\;\;\;\;\;\;\;\;\;\frac{\text{Cost PPIUD − Cost of Standard Practice}}{\text{Outcomes PPIUD −Outcomes of Standard Practice}}$$

**FIGURE 2:**
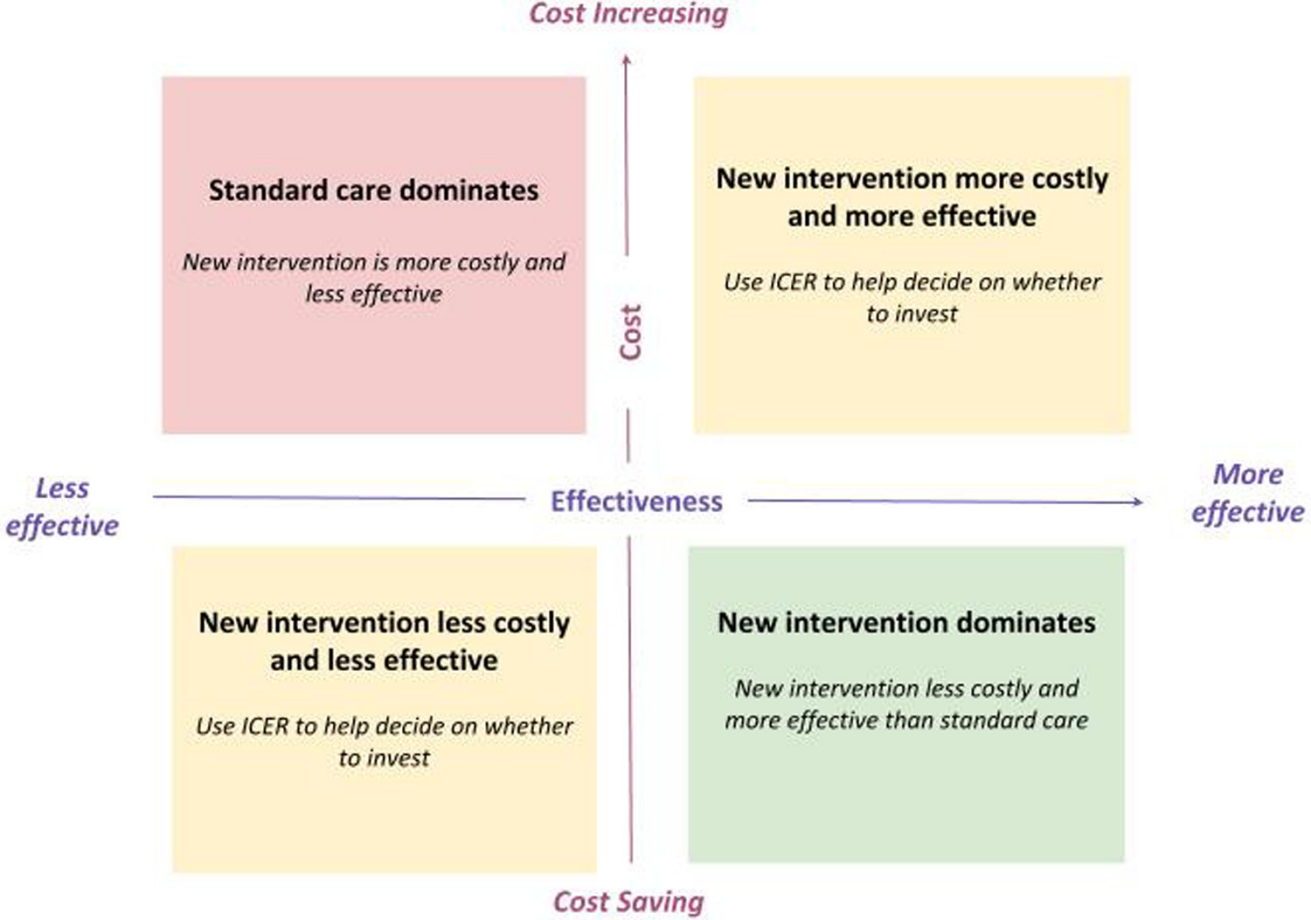
Cost-Effectiveness Plane Representing 4 Potential Outcomes of Cost-Effectiveness Analyses of Postpartum Intrauterine Device Initiative Abbreviation: ICER, incremental cost-effectiveness ratio. Source: Cost-effectiveness plane figure adapted from Cohen et al.^56^

The incremental costs and incremental benefits (outcomes) of the PPIUD initiative can be interpreted through a cost-effectiveness plane representing the 4 potential outcomes of the analyses (Figure 2).^56^

One-way sensitivity analyses were conducted to test the robustness of estimates included in the economic evaluations and describe the impact of uncertainty on parameter values (costs of direct service delivery and training costs, and varying the proportion of government reimbursements paid in Bangladesh).

## RESULTS

### Outcomes

In Bangladesh, the 6 participating facilities delivered 8,031 PPIUDs over the 36-month implementation period; in Tanzania the 6 participating facilities delivered 7,448 PPIUDs over the 27-month implementation period (Table 2).

**TABLE 2.** Results of Costing Analysis in Bangladesh and Tanzania

	Bangladesh		Tanzania	
	PPIUD Initiative	National Scale-Up Model	PPIUD Initiative	National Scale-Up Model
Program design				
Number of facilities^a^	6	36	6	28
Setup period, months	4	4	4	4
Implementation period, months	36	36	27	36
Number of PPIUDs inserted	8,031	26,507	7,448	43,928
Costing analysis				
Estimated total cost	US$539,285	US$1,979,140	US$1,869,507	US$6,910,494
Estimated cost of direct PPIUD service provision^b^	US$1.71	US$1.71	US$2.05	US$2.05
Cost per facility per year	US$27,986	US$17,373	US$130,697	US$79,223
Main cost driver	Facility staff^c^ (58% total cost)	Facility staff^c^ (53% total cost)	Training (76% total cost)	Training (80% total cost)
Estimated direct health care costs saved (Impact 2)	US$802,368	US$2,648,284	US$1,348,744	US$7,954,649
Estimated total costs after including estimated health care costs saved (Impact 2)	−US$263,083	−US$669,144	US$520,763	−US$1,044,156

Abbreviation: PPIUD, postpartum intrauterine device.

a Note the facilities included in the national scale-up model include the facilities in the PPIUD initiative plus additional facilities at the equivalent level of the public health care system. For Tanzania, each hospital in the scale-up model is assumed to have 4–6 associated satellite facilities that are trained in postpartum family planning counseling and given IEC materials to distribute and that refer clients to the hospitals, as was done in the PPIUD initiative.

b Includes cost of initial insertion, follow-up visit, and eventual removal using weighted averages. Cost of counseling is included for Tanzania but not for Bangladesh (cost of counselors in Bangladesh is included in staff costs, not direct service costs). Government reimbursements paid in Bangladesh are not included here.

c Facility staff in Bangladesh include counselors and honorariums in the PPIUD initiative. Counselors only are included in the national scale-up model.

### Service Provision and Total Cost

Table 2 displays the main results of the costing analysis. The direct service costs of a PPIUD include cost of insertion, follow-up visit, and removal. In Bangladesh, the counselors were employed full-time, so their costs were included in staff costs not direct services costs, whereas for Tanzania counseling was done by existing staff, so costs were calculated per PPIUD and are included here. The cost of direct service provision was estimated to be US$1.71 per PPIUD in Bangladesh (excluding government reimbursements) and US$2.05 in Tanzania. It was estimated that the reimbursement paid by the Government of Bangladesh (see above) would be paid 50% of the time during the implementation period, thus US$3.12 was added to the base cost, resulting in a cost per PPIUD with reimbursements included of US$4.83 in the Bangladesh analysis. In Bangladesh, the main cost driver was facility-level staffing, followed by national-level staffing. In Tanzania the main cost driver was training.

The direct health care costs saved by the PPIUD initiative, based on estimates from the Impact 2 tool, were US$802,368 in Bangladesh and US$1,348,744 in Tanzania (Table 2).

The direct health care costs saved by the PPIUD initiative were estimated to be US$802,368 in Bangladesh and US$1,348,744 in Tanzania.

### National Scale-Up Model

In the analysis for the national scale-up model, a 36-month implementation period was used for both countries. For Bangladesh it was estimated that the 36 facilities would deliver 26,507 PPIUDs, while for Tanzania it was estimated that the 28 facilities (plus 140 satellite facilities) would deliver 43,928 PPIUDs (Table 2). The analysis for the national scale-up model estimated direct health care costs saved of US$2,648,284 in Bangladesh, and US$7,954,649 in Tanzania (as estimated by the Impact 2 tool) (Table 2).

### Cost-Effectiveness

Table 3 displays ICER results for the PPIUD initiative presented both with and without the cost offset of the estimated direct health care savings from the Impact 2 tool.

**TABLE 3.** Cost-Effectiveness of PPIUD Initiative

Outcome of interest^a^	Bangladesh			Tanzania		
	Estimated Number	ICER Without Cost Offset^b^	ICER With Cost Offset	Estimated Number	ICER Without Cost Offset^b^	ICER With Cost Offset^c^
PPIUDs inserted	8,031	67.2	PPIUD dominates	7,448	251.1	69.9
CYPs	36,943	14.6	PPIUD dominates	34,261	54.6	15.2
Unintended pregnancies averted	16,683	32.3	PPIUD dominates	15,471	120.8	33.7
Maternal deaths averted	11	50,731.0	PPIUD dominates	30	62,316.9	17,358.8
Child deaths averted	63	8,613.0	PPIUD dominates	306	6,109.5	1,701.8
Total DALYs averted (maternal + child DALYs)	5,918	91.1	PPIUD dominates	27,626	67.7	18.9

Abbreviations: CYP, couple-years of protection; DALYs, disability-adjusted life years; ICER, incremental cost-effectiveness ratio; PPIUD, postpartum intrauterine device.

a Outcomes are the estimated service lifespan impacts from the Impact 2 tool.

b The ICER without cost offset is equivalent to the cost per outcome because the cost of standard practice is estimated as zero cost in both study groups without any impact on the ICER.

c When neither the intervention nor standard care “dominates,” the ICER should be used to decide whether or not to invest (see Figure 2).

In both countries, the PPIUD initiative was found to be more expensive and more effective than standard practice, before offsetting the direct cost savings to the health care system. In Bangladesh, the cost per outcome was estimated to be US$14.60 per CYP and US$91.13 per DALY averted, while in Tanzania the cost per outcome was estimated to be US$54.57 per CYP and US$67.67 per DALY averted compared with standard practice. When the cost offset generated from the Impact 2 tool was incorporated (from estimated direct cost savings to the health care system), in Bangladesh PPIUD “dominated” (i.e., PPIUD is cheaper and more effective). For Tanzania, the ICER with cost offset was estimated to be US$15.20 per CYP and US$18.90 per DALY averted compared with standard practice, meaning it remained more effective and more costly than standard care.

Table 4 displays ICER results for the national scale-up model. In Bangladesh, the cost per outcome was estimated to be US$16.23 per CYP and US$106.64 per DALY averted, while in Tanzania the results were estimated to be US$34.20 per CYP and US$43.31 per DALY averted. Once the estimated savings from direct health care costs averted were factored in (as estimated by the Impact 2 tool) PPIUD dominated for all outcomes in both countries, meaning that it would be both cheaper and more effective to provide the PPIUD intervention compared with standard care. Full results of the national scale-up model can be found in the Supplement.

**TABLE 4.** Cost-Effectiveness of National Scale-Up Model

Outcome of Interest^a^	Bangladesh			Tanzania		
	Estimated Number	ICER Without Cost Offset^b^	ICER With Cost Offset	Estimated Number	ICER Without Cost Offset^b^	ICER With Cost Offset
PPIUDs inserted	26,507	74.7	PPIUD dominates	43,928	157.31	PPIUD dominates
CYPs	121,932	16.2	PPIUD dominates	206,064	34.2	PPIUD dominates
Unintended pregnancies averted	55,062	35.9	PPIUD dominates	91,248	75.73	PPIUD dominates
Maternal deaths averted	18	107,057.9	PPIUD dominates	120	57,587.45	PPIUD dominates
Child deaths averted	207	9,576.2	PPIUD dominates	1,804	3,830.65	PPIUD dominates
Total DALYs averted (maternal + child DALYs)	18,558	106.6	PPIUD dominates	159,561	43.31	PPIUD dominates

Abbreviations: CYP, couple-years of protection; DALYs, disability-adjusted life years; ICER, incremental cost-effectiveness ratio; PPIUD, postpartum intrauterine device.

a Outcomes are the estimated service lifespan impacts from the Impact 2 tool.

b The ICER without cost offset is equivalent to the cost per outcome because the cost of standard practice is estimated as zero cost in both study groups without any impact on the ICER.

### Sensitivity Analyses for Cost Adjustments

**FIGURE 3:**
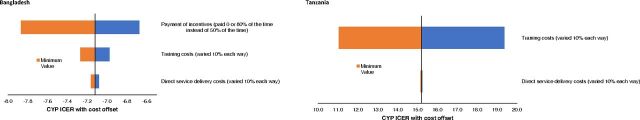
Sensitivity Analyses for Cost Adjustments Showing Incremental Cost-Effectiveness Ratio for Postpartum Intrauterine Device Initiative in Bangladesh and Tanzania Abbreviations: CYP, couple-years of protection; ICER, incremental cost-effectiveness ratio.

In Bangladesh, the ICER was most sensitive to the rate of payment of government reimbursements (Figure 3). With all the parameters and scenarios tested, the PPIUD intervention remained cheaper and more effective than standard practice, indicating it was the dominant strategy. In Tanzania, the ICER was most sensitive to variations in costs of training (Figure 3). For both scenarios tested, the PPIUD intervention remained highly cost-effective. The scenario with the highest cost per DALY was an increase of 10% of training costs, which resulted in a cost per DALY of US$72.83 before estimated health savings were factored in.

For the national scale-up analysis, the models were most sensitive to changes in rate of payment of government reimbursements (Bangladesh) and training costs (Tanzania). However, the models remained cheaper and more effective than standard care, indicating the PPIUD intervention was the dominant strategy in all scenarios tested. Details can be found in the Supplement.

### Sensitivity Analyses for Uptake of Alternate PPFP

**FIGURE 4:**
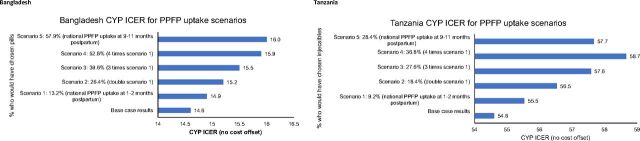
Sensitivity Analyses for Uptake of Alternate Postpartum Family Planning Methods During the Extended Postpartum Period in Bangladesh and Tanzania Abbreviations: CYP, couple-years of protection; ICER, incremental cost-effectiveness ratio; PPFP, postpartum family planning.

A number of scenarios were tested to estimate the effect on the ICER of different proportions of PPIUD adopters taking up an alternate family planning method during the extended postpartum period (see Figure 4). The scenarios tested were based on the national PPFP uptake rate at either 1–2 months or 9–11 months postpartum. Details of the different scenarios and assumptions made can be found in the Supplement.

For all scenarios in both countries, the PPIUD intervention remained more costly and more effective than standard care (before estimated direct cost savings to the health care system were factored in), and is likely cost-effective. Even in the most extreme scenarios (the 9–11 month PPFP uptake rate in Bangladesh, and 4 times the 1- to 2-month PPFP uptake rate in Tanzania), the ICER did not change substantially from the base case results (14.6 in Bangladesh, 54.6 in Tanzania). Details can be found in the Supplement.

## DISCUSSION

### Summary of Key Findings

The cost per CYP of the PPIUD initiative was US$14.60 in Bangladesh and US$54.57 in Tanzania before considering longer-term cost savings. In both countries, the PPIUD initiative was found to be more effective than standard PPFP practice. In Bangladesh, once the costs savings for the health care system were factored in, the PPIUD initiative was also found to be cheaper than standard practice. Despite the overall higher costs in Tanzania, cost per outcomes related to deaths averted and DALYs averted were less in Tanzania compared with Bangladesh because overall maternal health outcomes in Tanzania were much poorer,^39^ thus the estimated impact of averting a pregnancy was much greater. In both countries, when PPIUD insertion was modeled to national-level scale-up, the estimated direct health care savings to the government exceeded the estimated cost of rolling out PPIUD services. In other words, these analyses suggest that rolling out PPIUD services nationally would save costs in the long run.

International thresholds state that interventions that avert 1 DALY for less than the average per capita GDP for a given country are considered very cost-effective (see the limitations to these thresholds outlined below),^57^ while country-specific cost-effectiveness thresholds for Bangladesh and Tanzania range from 3% to 77% and from 4% to 86% of GDP per capita, respectively.^58^ Our cost per DALY estimates are cost-effective under all proposed thresholds. In Bangladesh, the cost per DALY averted was US$91.13 (5.4% of the 2018 GDP per capita of US$1,698), and in Tanzania the cost per DALY averted was US$67.67 (6.4% of the 2018 GDP per capita of US$1,501).

### Assumptions and Limitations

Due to the lack of direct comparability between immediate PPFP and family planning in the extended postpartum period, as well as the lack of necessary data, we did not factor into our analysis the proportion of PPIUD adopters who would otherwise have taken up an alternate PPFP method at a later date. As such, we may have overestimated the impact of the PPIUD initiative. We ran sensitivity analyses to test the impact of different proportions of women taking up alternate methods (see the Supplement for details). In all scenarios, when the estimated direct cost savings to the health care system from the PPIUD initiative were not factored in, the PPIUD initiative remained more expensive and more effective than standard practice and was likely cost-effective. The change in ICER was not substantial from our base ICER, suggesting only a small impact from women taking up alternate methods in the extended postpartum period. This outcome is because the most commonly used family planning methods in both countries are short-acting (and so lead to fewer CYPs) and are more expensive per CYP than the PPIUD.

Additional limitations of the evaluation include reliance on self-reported data and estimates for some measures, for example, time spent on the PPIUD initiative by project management staff and time spent on PPIUD delivery by clinical staff. We minimized reporting error by collecting multiple estimates, removing outliers, and reporting averages. We used sector standard CYP factors that do not account for services being delivered postpartum, when fertility may be lower than at other times due to abstinence or lactational amenorrhea,^51^ although this effect is dependent on women breastfeeding exclusively and only applies for the first 6 months postpartum.

The WHO guidance from 2001 to determine cost-effectiveness thresholds based on a country's per capita GDP has been criticized for not reflecting opportunity cost^58^ and lacking country specificity. It should be used alongside other country-specific information, such as the overall budget available for health.^59^ To address this issue, we also compared our findings with available country-specific thresholds (Summary of key findings above).

Certain costs, such as costs of demand generation activities and costs of treating complications, were not included in the analysis, which may have led to an underestimate of the true cost of scaling up PPIUD. Demand generation activities were not included because they were not part of the PPIUD initiative. Costs of treating complications were not included because there are insufficient data on the rate, type, and severity of PPIUD complications; however, these costs are not likely to be substantial and therefore likely would not impact the final analysis significantly. Similarly, there may have been additional benefits to the PPIUD initiative that were not captured in this evaluation. These possible benefits include increased uptake of other contraceptive methods during the immediate postpartum period and increased uptake of any contraceptive method after the immediate postpartum period due to improved PPFP counseling, uptake of PPIUD at nonparticipating facilities by providers trained through the initiative, and personal cost and time savings to women that take up a PPIUD. For the national scale-up model we needed to make several assumptions regarding costs and activities, which are described in full in the Supplement.

A further limitation of our analysis is that we only considered what was done in the PPIUD initiative, and this may differ if PPIUD rollout is run by the government or if the national context changes. For example, government-run PPIUD training might be longer than that used during the initiative, staff may already be in place and trained to provide PPFP counseling, and if different types of facilities were included, these would likely have different levels of uptake and costs. To explore these possibilities, we repeated the analyses with some adjustments to the intervention design. Details of this analysis and the results are in the Supplement.

### Comparison With Existing Literature

Although LARCs have been consistently demonstrated to be more cost effective than short-acting methods in high-income countries,^60^^,^^61^ there are few comparable studies on the cost-effectiveness of delivering postpartum contraception and even fewer specifically on immediate postpartum IUD.^62^ In addition, comparisons with other studies are of limited use due to different implementation approaches, different methodology for calculating cost, and different costs in different countries.

Previous studies comparing contraceptive methods have consistently found IUD to have a lower cost per CYP compared with other methods of contraception. One study in Kenya (not of postpartum contraception) reports an estimated cost of US$1.37 per CYP for IUD, US$1.60 for female sterilization, US$4.06–US$6.17 for implants, US$6.34 for IUS, US$6.88 for oral contraceptives, and US$7.07–US$12.47 for injectables (ranges represent different types of implant and injectables).^63^ A study from Rwanda reports an estimated cost of US$6 per CYP for PPIUD compared with US$21 per CYP for postpartum implant.^64^

The only known studies to have considered the cost-effectiveness of immediate PPIUD provision are Wall et al.^64^ in Rwanda and Washington et al.^65^ in the United States. The latter found that immediate PPIUD results in cost savings of US$282,540 per 1,000 women and a gain of 10 quality adjusted life years. Wall et al.^64^ used a micro-costing approach similar to our analyses to estimate the incremental cost of PPIUD and postpartum implants compared with standard methods, from the perspective of the health system, in Kigali, Rwanda. The authors included and excluded similar costs as our analyses, but unlike the PPIUD initiative, they conducted and included the costs of promotional activities. The resulting cost per PPIUD inserted was US$25 and cost per CYP for PPIUD was US$5, lower than the results in our analyses. However, the Rwanda initiative did include reimbursements paid directly to providers and community health workers referring women to providers and had a higher uptake rate of PPIUDs of 16% (compared with 5%–8% uptake in our analyses), making direct comparisons difficult.

### Significance of Results

Compared with previous analyses, our estimates of cost per PPIUD inserted and cost per CYP for PPIUD were generally higher than those reported in peer-reviewed publications, which could reflect our very detailed micro-costing approach as well as differing costs between countries. Nonetheless, our results indicate that even with these higher costs, national introduction and scale-up of PPIUD in Bangladesh and Tanzania are expected to be highly cost-effective or even cost saving. Both the cost-effectiveness and the impact of PPIUD may improve over time as some costs will decrease (for example, no repeat setup costs, and all facilities having trained providers in place), while the impact may increase as awareness and acceptability of the method improve among providers, women and their families, and communities. In addition, potential future national rollout of the PPIUD initiative may be positively affected by ongoing efforts in both countries to encourage births in facilities and improve the capacity of lower-level facilities, as well as efforts to increase awareness and availability of a range of postpartum contraceptive methods.^42^^,^^45^

National introduction and scale-up of PPIUD in Bangladesh and Tanzania are expected to be highly cost-effective or even cost saving.

While the PPIUD initiative was found to be cost-effective in both countries, the main cost drivers and actual costs differed. In Bangladesh, the largest cost driver was the staff employed at the facility level to counsel women on PPFP. Although more costly to the government, the inclusion of dedicated counselors was a highly effective way of providing quality counseling, which contributed to the success of the initiative,^19^ and has now been incorporated into the latest Bangladesh Costed Implementation Plan (2020–2022).^42^ Alternatively, this counseling role could be taken over by the new midwifery cadre of health worker, which could increase access to PPIUD services, while simultaneously reducing salary costs. In Tanzania, the main cost driver was training, partly because the training course was several days longer than in Bangladesh and also due to higher associated travel and meeting costs. PPIUDs in Tanzania are predominantly inserted by midwives, as opposed to doctors in Bangladesh,^66^ and so a longer training period was deemed necessary. The practice of frequently rotating providers to different clinical departments also meant that training had to be repeated frequently; the same challenge of high rotation of providers was also observed in a program to introduce PPIUD in Malawi.^67^ In the future, approaches such as on-the-job training could be used to reduce costs while maintaining quality, as has been demonstrated in other countries.^68^

FIGO shared the findings of the PPIUD initiative with the national societies of obstetricians and gynecologists and key government departmental heads in both countries, and they were received with much interest. In Bangladesh, there is an in-principle agreement to engage with the Obstetrical and Gynaecological Society of Bangladesh in the national rollout of PPIUD as part of a broader PPFP package, although the costs of this have not yet been ascertained. The Tanzanian government is currently seeking donor funding to progress national rollout of PPIUD. Furthermore, in both countries the PPIUD initiative has instigated changes to the preservice training curriculum of midwives and doctors; over time, this will lead to decreased need for detailed in-service training specifically for PPIUD provision.

It is estimated that making family planning widely accessible could reduce maternal mortality by one-third globally.^69^ As well as the health benefits arising from reduced risks to subsequent pregnancies, the newborn, and the wider family,^5^^–^^9^ there are additional benefits such as an increase in productivity and the economic value women can contribute to their societies when able to control their fertility.^70^^,^^71^ Offering immediate PPFP is an efficient way of giving women the choice to space or limit their pregnancies. Following the change in WHO MEC criteria, there are now more methods potentially available to women postpartum, each of which has advantages and disadvantages. Making available a broad contraceptive method mix allows women to choose the method most appropriate for them, increasing uptake and reducing the chances of discontinuation.^72^ The FIGO PPIUD initiative and many others have demonstrated that IUD insertion immediately postpartum is safe and feasible to implement.^29^ However, in practice many countries cannot consistently supply all LARC methods, and difficult cost-benefit decisions have to be made by governments when allocating resources. Information on cost-effectiveness can help guide government and policy resource allocation decisions to maximize value and impact. This economic evaluation estimated that from an implementation perspective, the provision of quality PPFP counseling and insertion of immediate PPIUD if chosen, is highly cost-effective in 2 LMICs, including when modeled to a national scale.

## CONCLUSION

The PPIUD initiative was found to be highly cost-effective in Bangladesh and Tanzania, with national scale-up of PPIUD estimated to produce long-term savings in health care costs. The true benefits to national governments are likely to be even greater than our analysis suggests owing to additional likely benefits not quantified. These analyses provide a compelling case for national governments and international donors to invest in the provision of quality contraceptive counseling before and around the time of delivery and for the routine inclusion of PPIUD within the suite of contraceptive methods made available during the immediate postpartum period in Bangladesh and Tanzania.

## Supplementary Material

20-00447-Eva-Supplement.pdf

## Notes

**Peer Reviewed**
