# Supplementary material for: Economic Evaluation of Provision of Postpartum Intrauterine Device Services in Bangladesh and Tanzania
Source: Glob Health Sci Pract. 2021 Mar 31;9(1):107–22. doi: 10.9745/GHSP-D-20-00447 (PMC8087427; doi:10.9745/GHSP-D-20-00447)
Supplement: 20-00447-Eva-Supplement.pdf [file 20-00447-Eva-Supplement.pdf]

Supplement to: Eva G, Gold J, Makins A, et al. Economic evaluation of provision of postpartum intrauterine device services in Bangladesh and Tanzania. *Glob Health Sci Pract*. 2021;9(1). https://doi.org/10.9745/GHSP-D-20-00447

Sensitivity Analysis to Test Impact of Alternate PPFP uptake

Introduction

Our evaluation assumes that the PPFP standard care is no PPFP uptake, in other words we assumed that all PPIUD adopters would *not* have taken up another PPFP method if the PPIUD had not been available. Although no other methods are offered *immediately* postpartum, it is likely that some (unknown) proportion of PPIUD adopters would in fact have taken up another family planning method during the extended postpartum period (up to one year). As a result, our analysis may overestimate the impact of the PPIUD intervention by underestimating the impact of standard PPFP care over the extended postpartum period.

Based on data from the 2011 DHS in Bangladesh and the 2010 DHS in Tanzania, the proportion of women using a method of family planning at one to two months postpartum is 13.2% in Bangladesh and 9.2% in Tanzania.[75] The proportion of women using a method of family planning at nine to eleven months postpartum is 57.9% in Bangladesh and 28.4% in Tanzania. The most popular modern FP method at three months postpartum was the pill in Bangladesh (61.5% of women using a modern FP method) and injectables in Tanzania (42.3% of women using a modern FP method).

Methods and results

Due to a lack of direct comparator, and a lack of data on: 1) what proportion of the PPIUD adopters would have taken up another method if the PPIUD had not been available, when and what method they would have taken up; 2) up-to-date data on PPFP uptake in the first year postpartum; and 3) cost data on other PPFP methods, we did not include the impact of uptake of a method after the

immediate postpartum period in our main analysis. Instead, we include here sensitivity analysis to test the impact of a range of potential scenarios of uptake of alternative PPFP methods on our base case results.

We have used the best available data estimates on PPFP uptake from the 2014 DHS Comparative Report No.36 *Use of Family Planning in the Postpartum Period*, which uses data from the 2011 Bangladesh DHS and the 2010 Tanzania DHS.[75] To estimate the proportion of PPIUD users who would have taken up another method, we used data on the proportion of women nationally who took up PPFP at one to two months postpartum. This assumes that the PPIUD adopters are typical of the general population in terms of likelihood of taking up PPFP when in fact they are probably *more likely* to be family planning users than the general population. To account for this, we tested a range of scenarios based on national PPFP uptake rates at one to month postpartum, plus the national PPFP uptake rate at nine to eleven months postpartum. The scenarios we included are described in Supplement Table 1.

Because we do not know what family planning method the PPIUD users might have taken up, we assumed all women used the most common PPFP method (pills in Bangladesh, injectables in Tanzania). Although the method mix has changed in the last decade in both countries, these are still the most popular family planning methods according to the most recent DHS in each country (2017-18 for Bangladesh and 2015-16 for Tanzania).

Table 1. Sensitivity Analysis for uptake of alternate postpartum family planning methods

Scenario Description	Bangladesh	Tanzania

Supplement to: Eva G, Gold J, Makins A, et al. Economic evaluation of provision of postpartum intrauterine device services in Bangladesh and Tanzania. *Glob Health Sci Pract*. 2021;9(1). https://doi.org/10.9745/GHSP-D-20-00447

		**Estimated % uptake of pill**	**ICER (with no cost offset)**	**Estimated % uptake of injectable**	**ICER (with no cost offset)**
	Base case result	0	14.6	0	54.6
1	The same proportion of women who take up PPFP nationally would have taken up an alternative method at 1-2 months postpartum (the most common method in each country)	13.2	14.9	9.2	55.5
2	Double uptake of scenario 1	26.4	15.2	18.4	56.5
3	Triple uptake of scenario 1	39.6	15.5	27.6	57.6
4	Four times uptake of scenario 1	52.8	15.9	36.8	58.7
5	The proportion of women using any FP method at 9-11 months postpartum	57.9	16.0	28.4	57.7

We have taken costs of the other methods from the best available sources. In Bangladesh we used the cost of pill provision (commodity plus personnel costs) published in the FP 2020 Bangladesh Costed Implementation Plan for the National Family Planning Programme 2016-2020.[76] In Tanzania we used the cost of injectable provision (commodity plus supplies plus personnel) from Guttmacher's Investing in Contraception and Maternal and Newborn Health, 2017 report.[55] In both cases the costing methodologies were similar to ours, and both are used by the national governments. We adjusted to 2018 USD using the same inflation adjustments made in the main analyses. Although we have used the best available costs, note that these are not specific to postpartum delivery and, for Tanzania, are regional rather than national costs. We present the ICER for CYP with no cost offset.

See Figure 4 in the article for the results of the sensitivity analysis. The base ICER for Bangladesh was 14.6 and for Tanzania it was 54.6. For all scenarios, the PPIUD intervention remained more costly and more effective than standard care, but likely still cost effective.

Conclusion

In all scenarios, the ICER does not increase substantially from our base ICER, even in the extreme scenario based on uptake of any method at nine to eleven months postpartum (Bangladesh) or four times the national PPFP uptake rate at one to two months postpartum (Tanzania). This is due to the fact that most family planning users in both countries use short-acting methods, which have fewer CYPs attributed and cost more per CYP than the PPIUD.

Supplement to: Eva G, Gold J, Makins A, et al. Economic evaluation of provision of postpartum intrauterine device services in Bangladesh and Tanzania. *Glob Health Sci Pract*. 2021;9(1). https://doi.org/10.9745/GHSP-D-20-00447

Setup and Implementation costs for the PPIUD Initiative in Bangladesh and Tanzania

Bangladesh

Table 2. Bangladesh Setup Costs (first 4 months)

**Item**	**Costs (2018 US$)**
Recruitment advertising costs	$61.72
National project staff costs for four months	$9,400.59
Initial meetings with facilities and stakeholders	$4,268.75
Purchase of fixed equipment for provision of PPIUD training and services	$3,145.13
Delivery of initial PPIUD training of trainers (TOT)	$9,833.73
Development of IEC materials, including any equipment needed	$5,592.08
**TOTAL COST** *Without overhead*	**$32,302.00**

Table 3. Bangladesh Implementation Costs (36 months)

**Item**	**Costs (2018 US$)**
Training of providers on PPIUD services, including refresher training	$34,841.07
Training of dedicated family planning counsellors, including refresher training	$4,823.25
National level project staffing costs	$84,605.32
Facility level staff costs - counsellors	$157,539.02
Facility level staff costs - honorariums	$125,747.18
Costs of clinical supervision	$4,442.23

Supplement to: Eva G, Gold J, Makins A, et al. Economic evaluation of provision of postpartum intrauterine device services in Bangladesh and Tanzania. *Glob Health Sci Pract*. 2021;9(1). https://doi.org/10.9745/GHSP-D-20-00447

Ongoing meetings with facilities and stakeholders	$1,989.10
Ongoing distribution costs of IEC materials	$439.05
Sharing of data and learnings	$4,736.64
**TOTAL COST**<br>*Without overhead*	**$419,162.86**

Supplement to: Eva G, Gold J, Makins A, et al. Economic evaluation of provision of postpartum intrauterine device services in Bangladesh and Tanzania. *Glob Health Sci Pract*. 2021;9(1). https://doi.org/10.9745/GHSP-D-20-00447

Tanzania

Table 4. Tanzania Setup Costs (first 4 months)

**Item**	**Costs (2018 US$)**
Recruitment advertising costs	$584.19
National project staff costs for four months	$18,392.18
Initial meetings with facilities and stakeholders	$12,601.48
Purchase of fixed equipment for provision of PPIUD training and services	$6,213.43
Delivery of initial PPIUD training of trainers (TOT)	$37,572.80
Development of IEC materials, including any equipment needed	$20,179.82
**TOTAL COST** *Without overhead*	**$95,543.90**

Table 5. Tanzania Implementation Costs (27 months)

**Item**	**Costs (2018 US$)**
Training of providers on PPIUD delivery, including refresher training	$663,298.74
Training of providers on PPFP counselling, including refresher training	$632,480.04
National level project staffing costs	$124,147.19
Facility level staff costs - honorariums	$44,917.15
Costs of clinical supervision	$22,830.60
Ongoing meetings with facilities and stakeholders	$12,267.98
Ongoing distribution costs of IEC materials	$13,882.06
Sharing of data and learnings	$74,934.12

Supplement to: Eva G, Gold J, Makins A, et al. Economic evaluation of provision of postpartum intrauterine device services in Bangladesh and Tanzania. *Glob Health Sci Pract*. 2021;9(1). https://doi.org/10.9745/GHSP-D-20-00447

**TOTAL COST**<br>*Without overhead*	**$1,588,757.86**

Supplement to: Eva G, Gold J, Makins A, et al. Economic evaluation of provision of postpartum intrauterine device services in Bangladesh and Tanzania. *Glob Health Sci Pract*. 2021;9(1). https://doi.org/10.9745/GHSP-D-20-00447

Details of National Scale-Up Model Design and Analysis

This section describes the design and results of the national scale-up model which was developed for each country to estimate the cost-effectiveness of PPIUD roll-out at the national level. The aim of this modeling exercise was to produce useful information on the estimated costs and impact of scaling up PPIUD nationally, if led locally, rather than by an international organization as was done for the FIGO PPIUD initiative reported in the main paper. The design of the national scale-up was based on what was considered to be the most likely next stage of PPIUD roll-out in each country, based on discussions with the country project teams and published national plans.[42,44]

Methods

The national scale-up model was based on the costs and outcomes of the PPIUD initiative, and the same costs were included. In Bangladesh, the scale-up was modeled to all 36 Government Medical College Hospitals nationally.[47] In Tanzania, the scale up model included all 28 Regional Referral Hospitals nationally,[48] as well as 140 satellite facilities (assuming 5 per hospital). The satellite facilities in Tanzania were assumed to play a similar role in PPFP counseling and referrals as they did during the PPIUD initiative. These scale-up facilities are broadly similar to the facilities included in the PPIUD initiative in terms of size and services offered, with the exception of 3 large facilities in Bangladesh included in the PPIUD initiative that account for a relatively high proportion of total national births. However, for the purpose of the scale-up analysis all facilities are assumed to provide similar numbers of births to each other.

We modeled national scale-up with a 4-month initial setup period (as was used in the PPIUD initiative) and a 36-month implementation period. For Tanzania, PPIUD insertion rates were based on the annual insertion rates during the PPIUD initiative, based on the assumption that the new

facilities are likely to be at the same baseline point as the facilities that participated in the PPIUD initiative, and are likely to increase their PPIUD uptake at a similar rate. In Bangladesh, PPPIUD insertion rates were based on those in the final 6 months of the PPIUD initiative (January to June 2018) as the country team reported there is now more awareness and acceptability of family planning in Bangladesh compared to 2015 when the PPIUD initiative started, suggesting a likely higher starting uptake rate.

In Bangladesh we used historical annual delivery data from the Government Medical College Hospitals to estimate the baseline (2018) number of deliveries and the annual increase in deliveries.[77] In Tanzania, national level data were not available so we used delivery data from the 6 facilities that were part of the PPIUD initiative to estimate the baseline (2018) and used projected annual increase in number of births to estimate the annual increase in deliveries.[78] We applied the estimated PPIUD uptake rates to this delivery data to estimate the number of PPIUDs that would be inserted each year during the national scale up model, and entered these into the Impact 2 tool for 2020-2022 to estimate the resulting impact.

Adjustments to the PPIUD initiative design.

For the national scale-up model, adjustments were made to the PPIUD initiative design, based on 1) how costs would differ if the roll-out were run by the national government and 2) lessons learned during the PPIUD initiative. The main adjustments for both countries were:

- Reduction of costs to align with expected government costs: no honorariums paid to existing facility level staff; reduction of costs associated with clinical supervision and meetings with facilities which would be incorporated into existing mechanisms;

- Central staff coordination costs were adjusted based on expected needs (some national coordination required as well as regional level coordination);
- No costs included for development of IEC and training materials, since these were developed under the PPIUD initiative (and thus don't need to be recreated).

The main country-specific adjustments were:

- **Bangladesh**: Assumption that 80% of government reimbursements for long term methods of contraception will be paid (vs 50% in the main analysis; country teams report that government reimbursements are now more regularly accessed), and addition of 2 hours of time to each training to allow for increased training on interpersonal communication;
- **Tanzania**: Refresher training delivered as 'on-the-job' supervision rather than a separate 1-day training (to minimize the costs and time of training). Insertion and counselling training were combined and reduced from 6 to 5 days to increase efficiency, as was done in the final phase of the PPIUD initiative.

Supplement to: Eva G, Gold J, Makins A, et al. Economic evaluation of provision of postpartum intrauterine device services in Bangladesh and Tanzania. *Glob Health Sci Pract*. 2021;9(1). https://doi.org/10.9745/GHSP-D-20-00447

Analysis

The analysis approach for the national scale-up model was the same as for the main analysis, with 2 additional parameters tested in sensitivity analysis. As well as adjusting direct service delivery and training costs +/-10%, we also adjusted national project management staff costs by +/-10% and tested the rate of payment of government reimbursements in Bangladesh at 0% and 80%.

We also re-ran our analysis with adjustments to the implementation design to examine how further changes to the design might affect the cost-effectiveness of rolling out PPIUD. The following design adjustments were tested:

- In Bangladesh, we estimated the effect of removing the initial training of counsellors and PPIUD trainers, to account for the potential scenario in which Government Medical College Hospitals already have trained counsellors and PPIUD trainers in place.

- In Tanzania, we estimated the effect of: a) extending the training length to align with the standard government PPFP training of 14 days; b) halving the number of providers trained, to account for the potential scenario in which there is less frequent rotation of providers between clinical departments; and c) expanding provision of PPIUD services to satellite facilities.

Results

Table 6 displays the estimated total cost and healthcare costs saved for the analysis of the national scale up model for Bangladesh and Tanzania. In both countries the estimated healthcare costs saved exceed the estimated total cost of rolling out PPIUD nationally.

Supplement to: Eva G, Gold J, Makins A, et al. Economic evaluation of provision of postpartum intrauterine device services in Bangladesh and Tanzania. *Glob Health Sci Pract*. 2021;9(1). https://doi.org/10.9745/GHSP-D-20-00447

Supplement to: Eva G, Gold J, Makins A, et al. Economic evaluation of provision of postpartum intrauterine device services in Bangladesh and Tanzania. *Glob Health Sci Pract*. 2021;9(1). https://doi.org/10.9745/GHSP-D-20-00447

Table 6. Results of Costing Analysis for National Scale-Up Model in Bangladesh and Tanzania

	Bangladesh	Tanzania
A. Estimated total cost of national scale up model	US$1,979,139.65	US$6,910,493.50
B. Estimated direct health care costs saved (Impact 2)	US$2,648,283.68	US$7,954,649.33
C. Estimated total costs after including estimated health care costs saved (Impact 2) (A - B)	-US$669,144.03	-US$1,044,155.83

Table 7 displays incremental cost-effectiveness ratio results for the national scale up model, presented both with and without the cost offset of the estimated direct healthcare savings. The national scale up model appears highly cost-effective in both countries. In Bangladesh the cost per outcome is estimated at $16.23 per CYP and $106.64 per DALY averted, while in Tanzania the results are estimated at $34.20 per CYP and $43.31 per DALY averted. Once the estimated savings from direct healthcare costs averted are factored in to the ICER calculations, the cost-effectiveness of PPIUD in the national scale up model ‘dominates’ for all outcomes in both countries, meaning that it would be both cheaper and more effective to provide the PPIUD intervention compared to standard care.

Supplement to: Eva G, Gold J, Makins A, et al. Economic evaluation of provision of postpartum intrauterine device services in Bangladesh and Tanzania. *Glob Health Sci Pract*. 2021;9(1). https://doi.org/10.9745/GHSP-D-20-00447

Table 7. Cost-Effectiveness of National Scale-up Model

	Bangladesh			Tanzania		
**Outcome of interest[1]**	**Estimated Number**	**ICER without cost offset[2]**	**ICER with cost offset**	**Estimated Number**	**ICER without cost offset[2]**	**ICER with cost offset**
PPIUDs inserted	26,507	74.7	PPIUD Dominates	43,928	157.31	PPIUD Dominates
CYPs	121,932	16.2	PPIUD Dominates	206,064	34.2	PPIUD Dominates
Unintended pregnancies averted	55,062	35.9	PPIUD Dominates	91,248	75.73	PPIUD Dominates
Maternal deaths averted	18	107,057.9	PPIUD Dominates	120	57,587.45	PPIUD Dominates
Child deaths averted	207	9,576.2	PPIUD Dominates	1,804	3,830.65	PPIUD Dominates
Total DALYs averted (maternal + child DALYs)	18,558	106.6	PPIUD Dominates	159,561	43.31	PPIUD Dominates

1 Outcomes are estimated service lifespan impacts from Impact 2 tool
2 The ICER without cost offset is equivalent to the cost per outcome because the cost of standard practice is estimated as zero cost in both study groups without any impact on the ICER

Sensitivity Analysis

As seen below in Figure 1, the models were most sensitive to changes in rate of government reimbursements (Bangladesh) and training costs (Tanzania). However, with all of the parameters and scenarios tested the PPIUD intervention remained cheaper and more effective than standard care, indicating it was the 'dominant' strategy.

Supplement to: Eva G, Gold J, Makins A, et al. Economic evaluation of provision of postpartum intrauterine device services in Bangladesh and Tanzania. *Glob Health Sci Pract*. 2021;9(1). https://doi.org/10.9745/GHSP-D-20-00447

Supplement to: Eva G, Gold J, Makins A, et al. Economic evaluation of provision of postpartum intrauterine device services in Bangladesh and Tanzania. *Glob Health Sci Pract*. 2021;9(1). https://doi.org/10.9745/GHSP-D-20-00447

Figure 1. Sensitivity analyses for national scale up model

Bangladesh

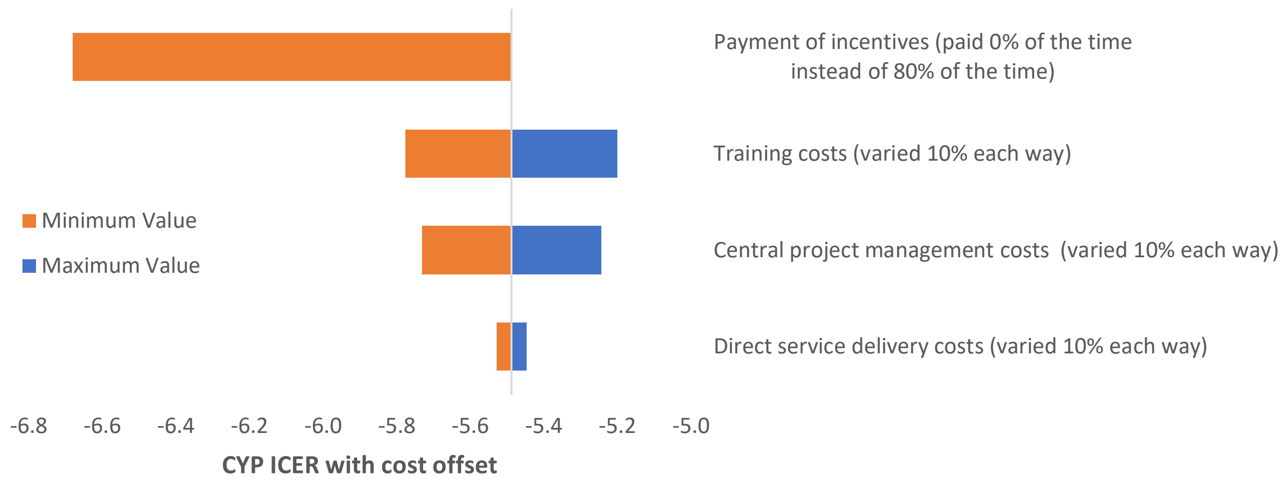

Tanzania

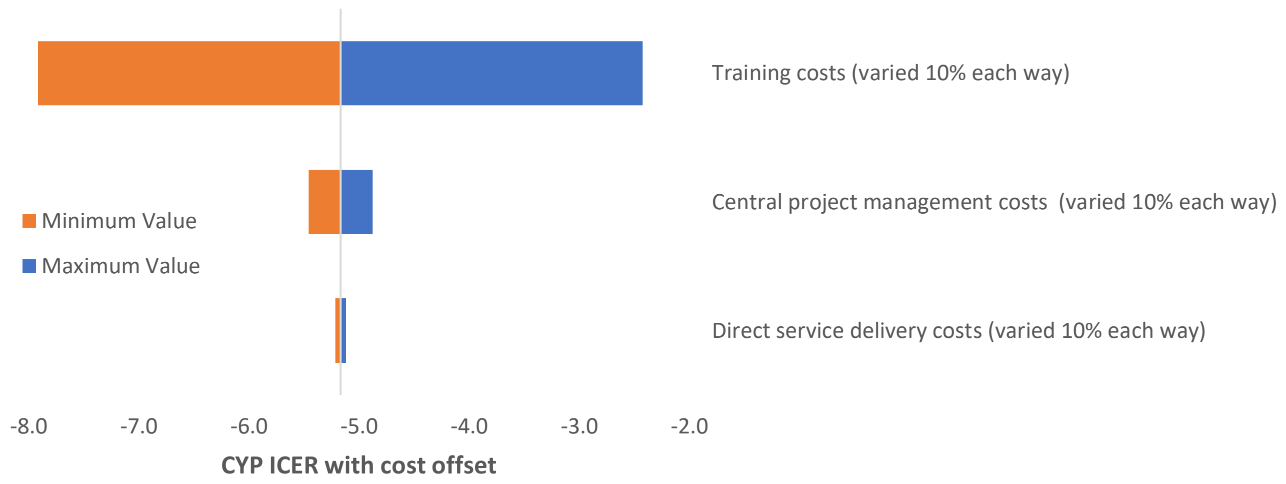

Design adjustments

In Bangladesh, removing the cost of the initial training of trainers in PPIUD insertion or removing the cost of the initial training of counsellors made minimal difference to the estimated total cost of implementation, or the resulting assessment of cost-effectiveness presented as ICERs.

In Tanzania, the key findings of the design adjustments were:

- Extending the training length to align with the standard government PPFP training of 14 days (vs 6 days in the PPIUD initiative and 5 days in the national scale-up model) resulted in a much higher cost, and the estimated direct healthcare savings no longer exceeded the total cost to implement. However, even with the extended training duration, cost per DALY averted is still below the 2018 per capita GDP of Tanzania of $1,051 and thus still considered very cost-effective (using internationally accepted thresholds that state that interventions that avert 1 DALY for less than average per capita GDP for a given country or region are considered very cost-effective).[57]

- Reducing the number of providers trained by half made a substantial reduction to the cost of the national scale up model (an estimated total cost of $4.1 million compared to $6.9 million).

- Expanding the services delivered at 4 satellite facilities to also include PPIUD insertion resulted in a 13% rise in cost for an estimated 5% additional PPIUDs provided. Expanding the services delivered at 105 satellite facilities to also include PPIUD insertion (75% of the total estimated number of satellite facilities) resulted in a 329% rise in cost for an estimated

38% additional PPIUDs provided. Due to a lack of data on which to base our projections, these estimates should be treated with caution.

Assumptions and limitations

In the national scale-up model we adjusted cost inputs to reflect how the initiative would be likely to be run if scaled up. However, we did not adjust the expected PPIUD insertion rates to account for any effect these changes to the design may have on PPIUD uptake. The projected number of PPIUD insertions were based on 2020, 2021 and 2022 projected data, but costs were maintained at 2018 levels, due to the difficulty of predicting future inflation rates and changes to item costs. We also removed honorariums paid to existing facility level staff, as these would not be paid if the initiative was led by the government. Not paying honorariums may have a negative effect on the motivation and commitment of the facility staff overseeing PPIUD introduction, which in turn could have a negative effect on PPIUD uptake. We assumed all scale-up facilities to be the same, despite differences in number of deliveries, in particular in the Bangladesh facilities.

Supplement to: Eva G, Gold J, Makins A, et al. Economic evaluation of provision of postpartum intrauterine device services in Bangladesh and Tanzania. *Glob Health Sci Pract*. 2021;9(1). https://doi.org/10.9745/GHSP-D-20-00447

REFERENCES

75. Winfrey W, Rakesh K. Use of Family Planning in the Postpartum Period: DHS Comparative Report. *ICF Int*. 2014;(36):3-44.

76. Ahmed S, Islam W. *Costed Implementation Plan for the National Family Costed Implementation Plan for the National Family Planning Program, Bangladesh 2016-2020*.; 2015.

77. Bangladesh Ministry of Health & Family Welfare. Bangladesh. Health Bulletin. 2018.

78. UNDP. World Population Prospects. Published 2019. https://population.un.org/wpp/
